# 32-week premature rupture of membranes caused by oropharyngeal microbiota

**DOI:** 10.1099/jmmcr.0.005121

**Published:** 2017-10-12

**Authors:** Alberto Hidalgo-Chicharro, Raquel Abad-Torreblanca, José María Navarro-Marí, José Gutiérrez-Fernández

**Affiliations:** ^1^​ Unidad de Gestión Clínica de Obstetricia y Ginecología, Hospital Universitario San Cecilio, Granada, Spain; ^2^​ Spanish Reference Laboratory for Meningococci, National Centre for Microbiology, Instituto de Salud Carlos III, Madrid, Spain; ^3^​ Servicio de Microbiología, Hospital Universitario Virgen de las Nieves-ibsGranada, Granada, Spain; ^4^​ Department of Microbiology, University of Granada-ibsGranada, Granada, Spain

**Keywords:** premature rupture of membranes, molecular characterization, *Staphylococcus aureus*, *Neisseria meningitidis*, *Haemophilus influenzae*, preventive antibiotic therapy

## Abstract

**Introduction.** Preterm premature rupture of membranes (PPROM) usually has a multifactorial etiology that is often unknown, although the most frequently reported cause is infection by group B *Streptococcus*. Therefore, the etiology of PPROM, although probably infectious, remains unknown in most cases. This case describes a PPROM caused by infection from oropharyngeal microbiota.

**Case presentation.** We report the case of a 26-yr-old pregnant woman. The gestational age was 32 weeks+5 days. Examinations in the emergency department revealed the release of clear amniotic fluid and a closed multiparous cervix with a length of 22 mm. Endocervical culture evidenced the growth of *Staphylococcus aureus*, serogroup B *Neisseria meningitidis* and *Haemophilus influenzae*.

**Conclusion.** Preventive antibiotic therapy should consider: opportunistic infections by normal genital microbiota, infections due to sexual activity, opportunist microorganisms derived from oral sex, and the hematogenous spread of oral bacteria.

## Abbreviations

PPROM, Preterm premature rupture of membranes; PROM, Premature rupture of membranes.

## Introduction

Premature rupture of membranes (PROM) is produced when amniotic membranes tear before labor onset and is recorded in around 8 % of full-term gestations [1]. Preterm premature rupture of membranes (PPROM) takes place before the 37th week of gestation, with an incidence of 2–4 % of pregnancies, and it is associated with higher maternal and perinatal morbidity and mortality, mainly related to infectious processes and prematurity [2]. Among maternal complications, which include postpartum infection, premature placental detachment, and maternal sepsis, we highlight clinical chorioamnionitis for its incidence and severity. In order of decreasing frequency, perinatal complications include respiratory distress, neonatal sepsis, intraventricular hemorrhage, necrotising enterocolitis, and neurological lesions [1]. Full-term PROM frequently has a physiological cause and is a consequence of uterine contractions [1]; however, PPROM usually has a multifactorial etiology that is often unknown, although the most frequently reported cause is infection, observed in up to 60 % of cases [3, 4]. Therefore, the etiology of PPROM, although probably infectious, remains unknown in most cases.

The obstetric approach varies as a function of gestational age, actively inducing the pregnancy in full-term PROM [5, 6] but performing an overall evaluation of maternal-fetal status in PPROM. In the latter situation, an assessment is made of the relative risks and benefits of a wait-and-see attitude versus pregnancy induction, considering signs of infection and/or prematurity and ordering antibiotic treatment when PPROM is diagnosed [7]. Multiple combinations of antimicrobial drugs have been proposed, and better perinatal and maternal outcomes have been reported for the prophylactic administration of some new combinations [8]. This study describes a case of PPROM caused by infection from oropharyngeal microbiota.

## Case report

We report the case of a 26-year-old pregnant woman of Arab ethnicity in her 32nd gestational week with a history of two previous full-term eutocic deliveries and no medical-surgical history of interest except for body mass index >30 kg m^−2^ and non-operated umbilical hernia. She did not smoke, drink alcohol, or use illicit drugs. She reported no drug allergies. She arrived at the emergency obstetrics and gynecology department due to the vaginal discharge of clear fluid (hydrorrhea), with no uterine contractions or bleeding. According to her clinical records, she had not previously undergone an ultrasonography examination during the pregnancy. The gestational age was estimated as 32 weeks+5 days by ultrasonic fetal biometry. The patient had normal blood pressure (126/79 mmHg) and a good general health status, with no fever (36.5 °C). Examination with a sterile speculum in the emergency department revealed the release of clear amniotic fluid, while transvaginal ultrasound showed a closed multiparous cervix with a length of 22 mm. Because of the clinical manifestation of amniorrhea, there was no need to apply techniques to confirm PPROM. Abdominal ultrasound showed the fetus in cephalic position with cardiac activity and active fetal movements, a normally inserted placenta on the upper anterior side, and a normal amount of amniotic fluid. The fetus weight estimated by the Hadlock formula was 2045 g, and the biometrics indicated 32+0 weeks of gestation. Urine, endocervical, and vaginal-rectal cultures were taken, and complete blood and urine analyses were performed. The cardiotocographic record at admission showed good fetal reactivity and the absence of uterine contractions.

Post-admission, the hospital protocol for PPROM was followed, administering antibiotherapy with intravenous ampicillin (2 g ampicillin then 1 g/6 h for 48 h) and oral azithromycin (1 g azithromycin in monodosis), withdrawing ampicillin after the first 48 h and then administering 500 g/8 h for 5 days completing the lung maturation regimen with betamethasone. The urine culture was negative, while the vaginal-rectal culture was positive for group B *Streptococcus* (GBS) (susceptible to ampicillin). Endocervical culture evidenced the growth of *Staphylococcus aureus (*susceptible to azithromycin), serogroup B *Neisseria meningitides (*susceptible to azithromycin), and *Haemophilus influenza* (susceptible to ampicillin). The *N. meningitidis* isolate was sent to the Neisseria Reference Laboratory of the Spanish National Microbiology Center for characterisation, confirming the isolation of *N. meningitidis* serogroup B by agglutination with specific monoclonal antibodies. Molecular characterisation of the strain included determination of the genosubtype by sequencing VR1 and VR2 variable regions of the external membrane protein PorA [9], and evaluation of the sequence type (ST) and clonal complex by Multilocus Sequence Typing (MLST) [10]. Molecular study of the isolate revealed a 7–2, 30 genosubtype strain belonging to the ST-53 clonal complex, which is frequently associated with nasopharyngeal carriage [11]. Screening for *Chlamydia trachomatis, Neisseria* *gonorrhoeae*, *Mycoplasma* spp., and *Ureaplasma* spp. yielded negative results. Although the patient remained clinically stable and afebrile, the labor was induced with intravenous oxytocin followed by eutocic delivery in gestation week 33+4 after the patient signed her informed consent. The motive for the induction was suspicion of subclinical chorioamnionitis, based on her increasing leukocytosis, neutrophilia, and C-reactive protein (CRP) levels since admission (leukocytosis of 11 030 at admission vs 14 650 at induction, neutrophilia of 83 % at admission: vs 81 % at induction, and CRP of 11.27 mg l^−1^ at admission vs 37 mg l^−1^ at induction). The CTG remained normal, with no uterine tenderness. The newborn was female and weighed 1970 g, with Apgar scores at 1 and 5 min of 9 and 10, respectively, umbilical artery pH of 7.32, and umbilical vein pH of 7.37, and she was admitted to the Neonatal Intensive Care Unit (NICU). During her NICU stay, the newborn remained clinically stable, with no need for vasoactive drugs or other advanced support measures, and she received prophylactic antibiotherapy in accordance with the hospital protocol. Cultures taken at admission and throughout the NICU stay were negative for infections. Transfontanelar ultrasound at admission ruled out intraventricular hemorrhage. The newborn was discharged at 13 days of life with a weight of 2180 g, cephalic perimeter of 32 cm and length of 47 cm, and no abnormal clinical findings.

## Discussion

PPROM of infectious etiology is an entity with potentially severe maternal-fetal morbidity and mortality and an added risk of prematurity. In the majority of cases, the pathogen derives from normal genital microbiota, but it can also be the consequence of unprotected sexual behavior (e.g. *Neisseria gonorrhoeae* or *Chlamydia trachomatis*). Our experience of epidemiologic changes in the etiology of PPROM means that a more complete diagnostic procedure must be followed, with wider analytic studies, adapting available diagnostic procedures to clinical needs. New culture-independent tools, including DNA amplification techniques have related several oral bacteria species to PPROM and chorioamnionitis and sometimes yield positive results even when vaginal and endocervical cultures are negative [12–15]. These new techniques can help to detect a possible unusual bacterial infection when vaginal and endocervical cultures are negative in cases of chorioamnionitis, preterm birth, or PPROM.

Among multiple antimicrobial combinations proposed for PPROM treatment, those based on ampicillin and/or cephalosporins have been the most widely used. Novel combinations were recently found to be efficacious and were reported to improve perinatal and maternal outcomes in comparison to the classic regimen [16, 17]. A meta-analysis in 2015 also proposed a prophylactic role for microbiotics in limiting the prolongation of pregnancy in PPROM; however, there is insufficient evidence to support the routine use of antibiotics during pregnancy to prevent infectious adverse effects, and some antibiotherapies may cause additional newborn morbidity [8].

The bacteria identified here are habitual colonising opportunistic pathogens. In the present patient, the isolation of oropharyngeal microbiota in endocervical culture suggested oral sex or hematogenous bacterial spread from the oral cavity as possible transmission mechanisms. Hence, preventive antibiotic therapy should consider: opportunistic infections from normal genital microbiota, infections due to sexual activity, opportunist microorganisms derived from oral sex, or hematogenous spread of oral bacteria.
